# Alternative iliofemoral revascularization in extensive aortoiliac occlusive disease

**DOI:** 10.1590/1677-5449.180083

**Published:** 2019-06-19

**Authors:** Júlio César Gomes Giusti, Janderson Ribeiro Fernandes, Samara Pontes Soares, Karoline Rayana dos Santos, Fabio Henrique Rossi, João Paulo Neves Beraldo, Francisco Cardoso Brochado

**Affiliations:** 1 Hospital Municipal Carmino Caricchio – HMCC, Departamento de Cirurgia Vascular, São Paulo, SP, Brasil.; 2 Instituto Dante Pazzanese de Cardiologia – IDPC-SP, Departamento de Cirurgia Vascular, São Paulo, SP, Brasil.

**Keywords:** atherosclerosis, arterial stenosis, autologous transplant, vascular graft

## Abstract

Over recent decades, there has been a considerable increase in use of endovascular methods to treat aortoiliac occlusive disease. It has been demonstrated that this approach offers many benefits, primarily for non-complex arterial lesions of the iliac axis, but difficulties persist with achieving adequate results over the medium and long term when treating extensive occlusive disease. Arterial bypasses to alternative vicarious arteries of the femoral-genicular complex for limb salvage are well known in the literature describing cases that are not favorable for conventional or endovascular surgery. We describe the case of a patient with extensive aortoiliac occlusive disease treated with an arterial bypass in the iliofemoral territory, using an alternative autologous substitute and the descending lateral femoral artery as recipient artery. Alternative bypasses and substitutes that are normally reserved for exceptional cases can and should be part of the vascular therapeutic arsenal and have a contribution to make in cases in which endovascular surgery does not yet enable us to achieve good results.

## INTRODUCTION

Over recent decades, endovascular treatment has achieved good results in stenosing lesions and short occlusions involving the aortoiliac segment, but, to date, results are still poor for extensive occlusive disease and in cases in which the common femoral artery is compromised.^1^^,^ 2 Currently, endarterectomy, iliofemoral arterial bypass, or even aortofemoral bypass, are the most widely performed procedures in these cases.^3^

Bypass to alternative collateral arteries in the lower limbs has already been well-described in the literature by Barral et al.,^4^ Brochado-Neto et al.,^5^ and De Luccia et al.,^6^^,^ 7 using the descending genicular artery and the sural genicular arteries. The latter are extremely important in patients with extensive femoropopliteal occlusion and no trunk arteries to receive outflow.^5^^,^ 6 With regard to substitutes, good quality autologous grafts are to be preferred over prosthetic (heterologous) substitutes because of the lower rates of infection of the surgical site and greater patency. Furthermore, autologous veins are more resistant to scenarios with high peripheral vascular resistance, as is the case when alternative collateral recipient arteries are used. In common with the great saphenous vein, which remains the first choice when available, upper limb veins have demonstrated good results as alternative substitutes.^7^

Infection of surgical vascular access at the groin is still of great concern to vascular surgeons, because of the potential for severe outcomes, including limb loss and death.^8^^,^ 9 The principal factors involved in complications are diabetes, obesity, and malnutrition. The choice of surgical access and intraoperative and postoperative care are therefore essential to achieving good results.

## PART I: CLINICAL SITUATION

**Figure 1:**
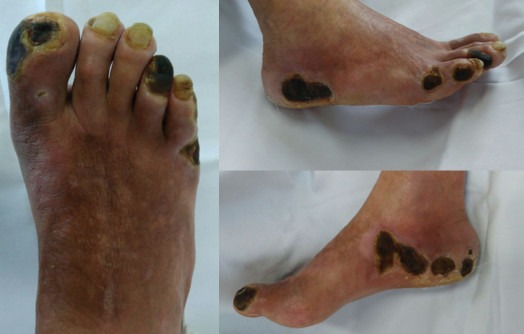
Necrotic lesions of the first, fourth, and fifth toes of the right foot and the medial and lateral surfaces of the right calcaneus.

**Figure 2:**
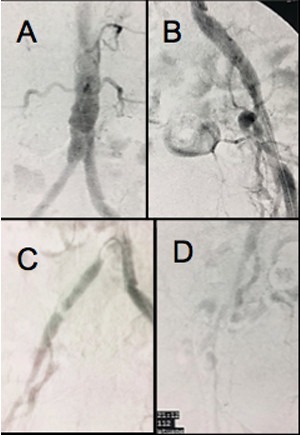
Aortic arteriography via retrograde left femoral puncture. (A) Origin of the iliac arteries free from stenosis; (B) Left iliac axis patent, only exhibiting mural irregularities; (C) Subocclusion of the distal third of the right common iliac artery; (D) Right external iliac with gradually reducing diameter and occlusion in the distal third. Ipsilateral common femoral artery occluded.

**Figure 3:**
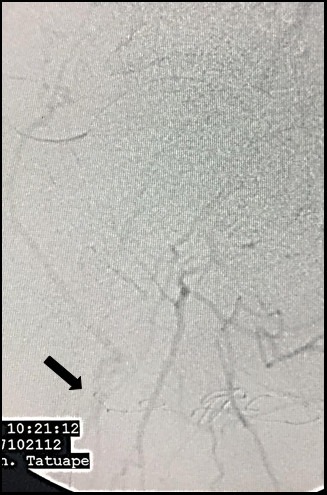
Arteriography demonstrating late re-filling of the descending lateral femoral artery (black arrow), via the iliofemoral collateral network.

The patient was a white, male, 58-year-old smoker with hypertension and diabetes who presented at our first-aid station complaining of pain at rest in the right lower limb, which, on physical examination was found to have necrosis of the first, fourth, and fifth toes, in addition to necrotic plaques on the medial and lateral surfaces of the calcaneus (Figure 1). His abdomen was convex, painless, and free from pulsatile masses. Palpation of pulses revealed total absence of right lower limb pulses and a full femoral pulse present in the left lower limb (3+/3). The ankle-brachial index (ABI) for the affected limb was calculated as 0.35 for the posterior tibial artery. Initial laboratory tests only revealed leukocytosis of 13,200/mm^3^, with no shift. Renal function was preserved, with serum creatinine of 0.8 mg/dL and urea of 29 mg/dL. Initially, we decided to perform aortic arteriography (Figure 2) of the right lower limb via a retrograde contralateral puncture, which showed a patent left iliac axis free from stenosis. The infrarenal aorta exhibited mural irregularities, but the origins of the iliac arteries were conserved. In the right iliac axis, there was distal subocclusive stenosis of the common iliac artery. The external iliac artery was patent at its origin, with gradual reduction in diameter and distal occlusion. The common, superficial, and deep femoral arteries were occluded. During the late phase of fluoroscopy, considerable collateralization was observed in the femoral territory, converging to the first branch of the deep femoral artery, in other words, the lateral circumflex femoral artery (Figure 3). Because of the extent of the occlusion, there was no contrast uptake in the more distal segments.

The patient was then examined with vascular ultrasound, in order to obtain greater information on femoral outflow and potential availability of autologous substitutes. Ultrasound confirmed occlusion at the iliofemoral transition and also of the ipsilateral femoral arteries, including the deep femoral artery, up to the third portion. The examination also showed refilling, via collaterals, of the descending lateral femoral artery, which was providing collateral supply, with 2.0 mm luminal diameter and monophasic “tardus-parvus” flow. Venous mapping only identified veins with adequate diameters in the left upper limb. The ipsilateral great saphenous vein was not adequate. Cardiological assessment classified surgical risk as intermediate (Lee-Vasc and ACP Classification).

In view of this presentation, a number of treatment options were discussed:

1 - High transfemoral amputation;
2 - Open endarterectomy of the right common iliac artery;
3 - Arterial bypass from the left external iliac artery to the descending lateral femoral artery with an alternative autologous substitute.

## PART II: WHAT WAS DONE

**Figure 4:**
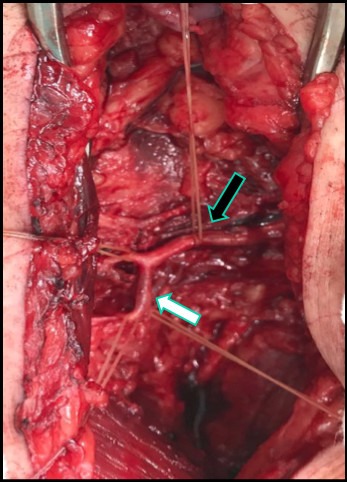
Surgical access via the right groin, revealing the lateral circumflex femoral artery (black arrow) and its descending branch, also known as the anastomotica magna (white arrow).

**Figure 5:**
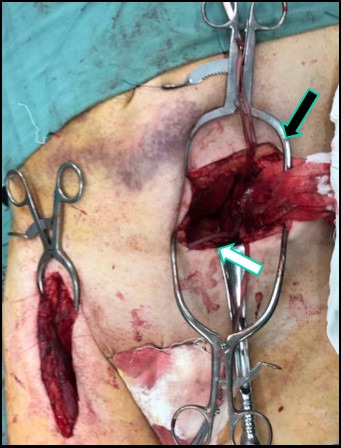
Retroperitoneal surgical access to the left external iliac (black arrow), showing venous graft following a suprapubic subcutaneous tunnel (white arrow).

**Figure 6:**
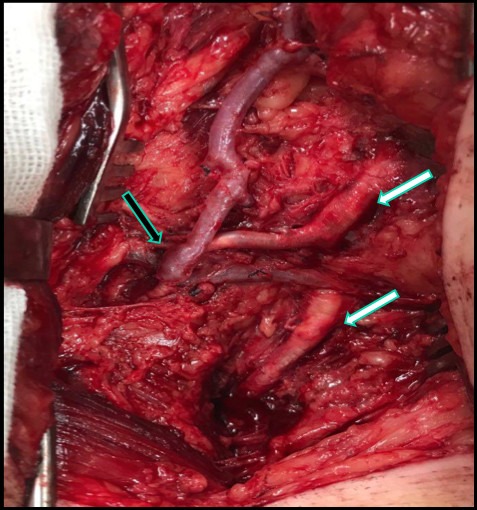
Surgical access via the right groin, showing the occluded first and second portions of the deep femoral artery (white arrows) and distal anastomosis to the descending lateral femoral artery (black arrow) with a venous substitute from the left upper limb.

**Figure 7:**
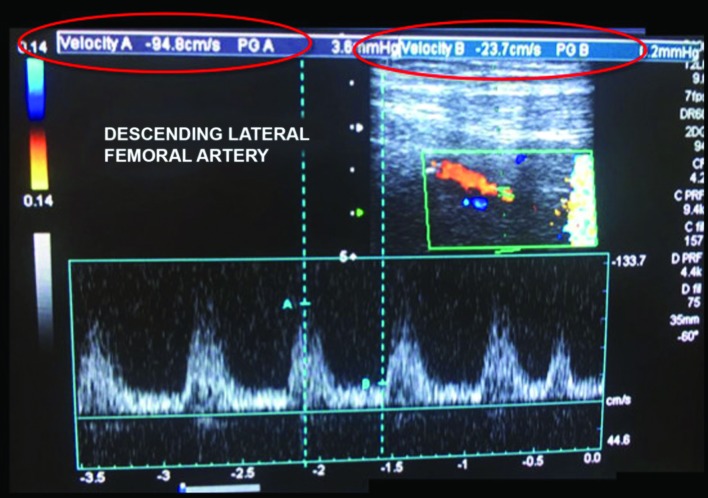
Postoperative follow-up control ultrasound at 5 months, showing adequate outflow via the descending lateral femoral artery, with biphasic wave, normal acceleration, and good amplitude.

We decided to perform a crossover bypass from the left external iliac artery to the right descending lateral femoral artery using an autologous substitute from the left upper limb. The procedure was conducted by two surgical teams simultaneously, under magnification with surgical loupes. The procedure was conducted in an operating theater, under general anesthesia. We started by dissecting the right groin, using a longitudinal incision, and dissected the deep femoral artery up to its third portion, confirming its total occlusion. We also identified occlusion of the lateral circumflex femoral artery, which is often the first perforating branch from the deep femoral artery. The descending lateral femoral branch, also known as the anastomotica magna, was patent, providing collateral circulation, with a fibroelastic appearance, and free from calcifications (Figure 4). Retroperitoneal access to the left external iliac artery was obtained via an arch-shaped incision at the iliac fossa, with no complications. This artery was conserved, fibroelastic, and exhibited low-level calcification. The venous graft was placed along a subcutaneous suprapubic tunnel. Concurrently, the other vascular surgery team explored the left upper limb to prepare the venous substitute. The basilic, superficial cubital, and median antebrachial veins had adequate diameters and consistency. It was observed that there was a nacreous venous thickening in the region of the cubital fossa, suggestive of prior thrombophlebitis. This segment was resected and a vein-to-vein anastomosis was constructed between the veins using two wire suture of 7-0 polypropylene. After systemic heparinization with 5,000 UI of unfractionated heparin, the proximal anastomosis to the left external iliac artery was constructed with two wires suture of 6-0 polypropylene. The vein was devalved, with continuous flow, using a Mills valvulotome. After the venous graft had been guided through the suprapubic subcutaneous tunnel (Figure 5), the distal anastomosis to the proximal segment of the descending lateral femoral artery was constructed, using a single wire suture of 7-0 polypropylene (Figure 6). After release of clamps, the segment caudal of the distal anastomosis had a wide pulse, suggestive of technically successful anastomoses. The patient was administered 25 mg of protamine hydrochloride to achieve partial reversal of the effects of the heparin. Incisions were closed and the patient was transferred to the Intensive Care Unit (ICU) and extubated, where he remained hemodynamically stable. The patient recovered satisfactorily and was discharged from the ICU to the wards on the second day after the operation. On the tenth day, the necrotic lesions underwent debridement, with good results, and he was discharged to outpatients follow-up on the fifteenth day after the operation. At discharge, ABI for the posterior tibial artery was 0.6. The arterial bypass was evaluated before hospital discharge and at 5 months’ follow-up, finding the graft patent, with good amplitude biphasic flow, and without stenosis (Figure 7). The luminal diameter of the anastomotica magna artery after the distal anastomosis was 2.6 mm.

**Figure 8:**
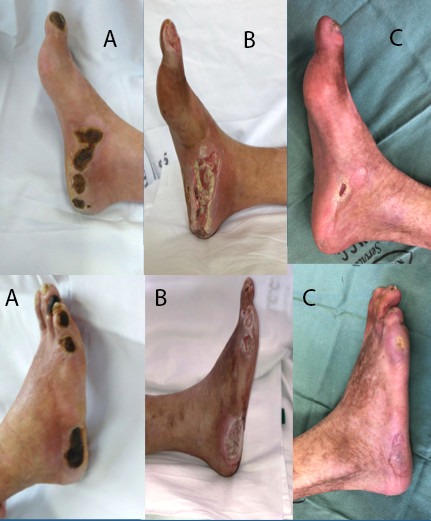
Progress of healing of right foot lesions. (A) Preoperative; (B) 10 days after operation; (C) 8 months after operation.

At the time of writing, the patient has been in follow-up for 8 months, is asymptomatic and independent and the lesions on his right foot have almost completely healed (Figure 8).

## DISCUSSION

Percutaneous transluminal angioplasty (PTA) is not always available in Brazilian hospitals and for younger patients, as was the case of the patient described here, the open technique offers better results over the long term. Furthermore, recanalization of the external iliac artery, which is sometimes associated with disease of the common femoral artery, can be extremely complex.^1^^,^ 2 When PTA is not acceptable for iliac lesions, conventional surgery is the most appropriate procedure to employ.^10^^,^ 11

In the case presented here, the primary reason for choosing the surgical approach was the extent of occlusive disease, which had compromised the entire iliofemoral transition. The choice to use a surgical access for the proximal anastomosis, using the left external iliac artery as the donor artery, was because our service has higher infection rates when the groin is used, possibly related to the number of comorbidities and low socioeconomic status of our patients.^8^ Groin infection rates after vascular procedures can reach 3 to 44%, according to the literature, with potential for serious outcomes, including increased mortality and limb loss.^9^^,^ 12 With regard to the substitute chosen, we preferred to use autologous venous material from the upper limb because of better compatibility of anastomotic diameters and low infection rates.^13^ In the case described here, the choice of a combination of veins from the upper limb was made on the basis of the inadequacy of the great saphenous vein, the venous thickening in the cubital topography, and our service’s considerable experience with using upper limb venous substitutes. Devalving of the venous substitute is the preferred option at our service because it preserves the anatomic profile of the native arteries in terms of their proximal and distal diameters, enabling more compatible anastomoses, and also preserves arterial phasicity within the graft.

The descending lateral femoral artery is known to play an important role in supragenicular and infragenicular collateral supply in femoropopliteal occlusions via natural communication with the genicular arteries and in this case it was the only viable option identified that could serve as recipient artery for the arterial bypass. This use has not yet been described in the literature, but similar procedures with alternative vicarious arteries, such as the descending genicular artery and the sural genicular arteries, are good bypass options in cases in which possible substitutes are limited, or in cases in which we cannot identify adequate trunk arteries to use as recipients.^7^^,^ 14

One possible criticism of our conduct with relation to this procedure could be the possibility of performing a hybrid treatment using PTA for the lesion of the right common iliac with an arterial bypass to the anastomotica magna artery, thereby avoiding a crossover graft. However, the necessary endovascular material was not available when this patient was admitted.

Alternative bypasses and substitutes that are normally reserved for exceptional cases can and should be part of the vascular therapeutic arsenal and have a contribution to make in cases in which endovascular surgery does not yet enable us to achieve good results.^4^^,^ 5 ^,^ 7 ^,^ 15 ^,^ 16
