# Supplementary material for: Alternative iliofemoral revascularization in extensive aortoiliac occlusive disease
Source: J Vasc Bras. 2019 Jun 19;18:e20180083. doi: 10.1590/1677-5449.180083 (PMC6637000; doi:10.1590/1677-5449.180083)
Supplement: Translation

# Translation: Revascularização ilíaco-femoral alternativa em doença oclusiva aortoilíaca extensa

**Authors:** Júlio César Gomes Giusti, Janderson Ribeiro Fernandes, Samara Pontes Soares, Karoline Rayana dos Santos, Fabio Henrique Rossi, João Paulo Neves Beraldo, Francisco Cardoso Brochado

**Affiliations:** 1 Hospital Municipal Carmino Caricchio – HMCC, Departamento de Cirurgia Vascular, São Paulo, SP, Brasil.; 2 Instituto Dante Pazzanese de Cardiologia – IDPC-SP, Departamento de Cirurgia Vascular, São Paulo, SP, Brasil.

**Keywords:** aterosclerose, estenose arterial, transplante autólogo, enxerto vascular

## Abstract

Nas últimas décadas, observou-se o aumento expressivo do tratamento endovascular para a doença oclusiva aortoilíaca. Muitos benefícios foram demonstrados, principalmente nas lesões arteriais não complexas do eixo ilíaco; porém, ainda existe dificuldade na obtenção de resultados adequados a médio e longo prazo quando falamos de doenças oclusivas extensas. As derivações arteriais para artérias vicariantes alternativas do complexo femoro-genicular para salvamento de membro são bem conhecidas na literatura em casos desfavoráveis para cirurgias convencionais ou endovasculares. Descrevemos o caso de um paciente portador de doença oclusiva aortoilíaca extensa tratado com derivação arterial no território ilíaco-femoral, utilizando substituto autógeno alternativo e a artéria descendente femoral lateral como artéria receptora. Derivações e substitutos alternativos, utilizados normalmente em casos de exceção, podem e devem fazer parte do arsenal terapêutico vascular, podendo contribuir nos casos em que a cirurgia endovascular ainda não nos permite alcançar bons resultados.

## INTRODUÇÃO

Nas últimas décadas, a terapia endovascular alcançou bons resultados em lesões estenosantes e oclusões curtas do segmento aortoilíaco, porém apresenta ainda hoje resultados limitados nas doenças oclusivas extensas ou naqueles casos em que a artéria femoral comum encontra-se acometida^1^^,^ 2. Atualmente, endarterectomia, derivação arterial ilíaco-femoral, ou até mesmo derivação aortofemoral, são os procedimentos mais realizados nesses casos^3^.

A derivação para artérias vicariantes alternativas de membros inferiores já foi bem descrita na literatura por Barral et al.^4^, Brochado-Neto et al.^5^ e De Luccia et al.^6^^,^ 7, em artéria genicular descendente e artérias geniculares surais. Estas últimas são de extrema importância em pacientes com oclusão femoropoplítea extensa e ausência de artérias receptoras tronculares^5^^,^ 6. Em relação aos substitutos, os enxertos autógenos de boa qualidade são os preferidos em relação aos substitutos protéticos (heterólogos), devido às menores taxas de infecção de sítio cirúrgico e maior perviedade. Além disso, as veias autógenas são mais resistentes a regimes de alta resistência vascular periférica, como é o caso de artérias receptoras alternativas e vicariantes. Assim como a veia safena magna, que ainda é a primeira escolha quando disponível, as veias de membros superiores já demonstraram bons resultados como substitutos alternativos^7^.

A infecção do acesso cirúrgico vascular em prega inguinal ainda é temida pelos cirurgiões vasculares, devido aos seus potenciais desfechos graves, como perda de membro e óbito^8^^,^ 9. Como principais fatores de complicação encontram-se diabetes, obesidade e desnutrição. Logo, a escolha cuidadosa do acesso cirúrgico e os cuidados intra e pós-operatórios são essenciais para alcançar bons resultados.

## PARTE I: SITUAÇÃO CLÍNICA

Paciente masculino, 58 anos, branco, tabagista, hipertenso e diabético, deu entrada no pronto atendimento de nosso serviço queixando-se de dor em repouso no membro inferior direito, onde apresentava, ao exame físico, lesões necróticas em primeiro, quarto e quinto pododáctilos, além de placas necróticas nas faces medial e lateral do calcâneo (Figura 1). O abdome era globoso, indolor, sem massas pulsáteis. À palpação dos pulsos, notou-se ausência global de pulsos em membro inferior direito e o pulso femoral presente e cheio em membro inferior esquerdo (3+/3). Foi realizado o índice tornozelo-braço (ITB) no membro acometido, com valor de 0,35 em artéria tibial posterior. Os exames laboratoriais de entrada acusavam apenas uma leucocitose de 13.200/mm^3^ sem desvio. A função renal estava preservada, com valores séricos de creatinina de 0,8 mg/dL e ureia de 29 mg/dL. Optamos inicialmente pela aortoarteriografia (Figura 2) de membro inferior direito através de punção retrógrada contralateral, que evidenciou um eixo ilíaco esquerdo pérvio e sem estenoses. A aorta infrarrenal apresentava irregularidades murais, porém as origens das artérias ilíacas estavam preservadas. No eixo ilíaco direito, evidenciou-se estenose suboclusiva distal em artéria ilíaca comum. A artéria ilíaca externa encontrava-se pérvia em sua origem, com redução gradual de seu diâmetro e oclusão distal. As artérias femorais comum, superficial e profunda estavam ocluídas. Em tempo tardio de fluoroscopia, observou-se colateralização importante em território femoral, convergente para o primeiro ramo da artéria femoral profunda, ou seja, a artéria circunflexa femoral lateral (Figura 3). Devido à extensão da oclusão, não houve contrastação dos segmentos mais distais.

**Figura 1:** Lesões necróticas em primeiro, quarto e quinto pododáctilos direitos, e nas faces medial e lateral do calcâneo direito.

**Figura 2:** Aortoarteriografia por punção retrógrada femoral esquerda. (A) Origem de artérias ilíacas sem estenoses; (B) Eixo ilíaco esquerdo pérvio apenas com irregularidades murais; (C) Suboclusão em terço distal de artéria ilíaca comum direita; (D) Ilíaca externa direita com redução gradual de diâmetro e oclusão em terço distal. Artéria femoral comum ipsilateral ocluída.

**Figura 3:** Arteriografia demonstrando reenchimento em tempo tardio de artéria descendente femoral lateral (seta preta), através de rede colateral ilíaco-femoral.

O paciente passou por ecografia vascular, na tentativa de obtermos maiores informações em relação ao escoamento femoral e aos potenciais substitutos autógenos disponíveis. O ultrassom confirmou a oclusão na transição ilíaco-femoral e também nas artérias femorais ipsilaterais, incluindo a artéria femoral profunda, até sua terceira porção. O exame também evidenciou o reenchimento, através de colaterais, da artéria descendente femoral lateral, que se encontrava vicariante, com diâmetro luminal de 2,0 mm e fluxo monofásico “tardus-parvus”. No mapeamento venoso, encontramos veias de diâmetros adequados apenas em membro superior esquerdo. A veia safena magna ipsilateral era inadequada. A avaliação cardiológica determinou risco cirúrgico intermediário (Classificação Lee-Vasc e ACP).

Diante desse quadro, algumas opções terapêuticas foram discutidas:

1 - Amputação transfemoral alta;
2 - Endarterectomia a céu aberto de artéria ilíaca comum direita;
3 - Derivação arterial da artéria ilíaca externa esquerda para artéria descendente femoral lateral com substituto autógeno alternativo.

## PARTE II: O QUE FOI FEITO

Optamos pela derivação cruzada da artéria ilíaca externa esquerda para a artéria descendente femoral lateral direita utilizando substituto autógeno de membro superior esquerdo. O procedimento foi realizado por duas equipes cirúrgicas, de forma simultânea e com magnificação através de lupas cirúrgicas. O procedimento foi realizado em centro cirúrgico, sob anestesia geral. Iniciamos pela dissecção da prega inguinal direita, através de incisão longitudinal, e dissecção da artéria femoral profunda até sua terceira porção, confirmando-se sua oclusão completa. Identificamos a oclusão também da artéria circunflexa femoral lateral, frequentemente primeira perfurante da artéria femoral profunda. O ramo descendente femoral lateral, também conhecido por anastomótica magna, encontrava-se pérvio, vicariante, com aspecto fibroelástico e sem calcificações (Figura 4). O acesso retroperitoneal à artéria ilíaca externa esquerda foi realizado através de incisão arciforme de fossa ilíaca, sem intercorrências. A artéria encontrava-se preservada, fibroelástica e com pouca calcificação. O trajeto do enxerto venoso foi subcutâneo suprapúbico. Concomitantemente, a outra equipe vascular explorava o membro superior esquerdo para preparo do substituto venoso. As veias basílica supracubital e intermédia de antebraço apresentavam-se com diâmetro e consistência adequados. Em topografia de prega cubital, notou-se um espessamento venoso de coloração nacarada, sugestivo de tromboflebite prévia. O segmento alterado foi ressecado e confeccionou-se uma anastomose veno-venosa entre as veias descritas com dois fios de polipropileno 7-0. Após heparinização sistêmica com 5.000 UI de heparina não fracionada, confeccionou-se a anastomose proximal em artéria ilíaca externa esquerda, utilizando dois fios de polipropileno 6-0. A veia foi devalvulada, sob fluxo contínuo, com auxílio de valvulótomo de Mills. Após passagem de enxerto venoso através de túnel subcutâneo suprapúbico (Figura 5), a anastomose distal foi confeccionada em segmento proximal de artéria descendente femoral lateral, utilizando fio único de polipropileno 7-0 (Figura 6). Após desclampeamento, o segmento caudal à anastomose distal apresentava pulso amplo, sugerindo sucesso técnico das anastomoses. Foram administrados 25 mg de cloridrato de protamina para reversão parcial do efeito heparinoide. As incisões foram fechadas e o paciente foi encaminhado à Unidade de Terapia Intensiva (UTI), extubado, e permaneceu estável hemodinamicamente. O paciente evoluiu satisfatoriamente e teve alta da UTI para enfermaria no segundo dia pós-operatório, e no 10° dia passou por desbridamento das lesões necróticas, com boa evolução, sendo liberado para acompanhamento ambulatorial no 15° dia de pós-operatório. O ITB de alta foi de 0,6 em artéria tibial posterior. A vigilância da derivação arterial foi realizada antes da alta hospitalar e com 5 meses de acompanhamento, e demonstrou enxerto pérvio, com fluxo bifásico de boa amplitude, sem estenoses (Figura 7). O diâmetro luminal da artéria anastomótica magna, após a anastomose distal, foi de 2,6 mm.

**Figura 4:** Acesso cirúrgico em prega inguinal direita evidenciando artéria circunflexa femoral lateral (seta preta) e seu ramo descendente, também conhecido como anastomótica magna (seta branca).

**Figura 5:** Acesso cirúrgico retroperitoneal à ilíaca externa esquerda (seta preta) evidenciando enxerto venoso em trajeto subcutâneo suprapúbico (seta branca).

**Figura 6:** Acesso cirúrgico em prega inguinal direita evidenciando artéria femoral profunda ocluída em suas primeira e segunda porções (setas brancas) e anastomose distal em artéria descendente lateral femoral (seta preta) com substituto venoso de membro superior esquerdo.

**Figura 7:** Vigilância ecográfica pós-operatória com 5 meses de evolução demonstrando escoamento adequado em artéria descendente lateral femoral, com onda de caráter bifásico, tempo de aceleração normal e boa amplitude.

O paciente encontra-se no momento deste relato com 8 meses de evolução, assintomático, independente, com cicatrização quase completa (Figura 8) das feridas em pé direito.

**Figura 8:** Evolução cicatricial das feridas de pé direito. (A) Pré-operatório; (B) 10º dia de pós-operatório; (C) 8 meses de pós-operatório.

## DISCUSSÃO

A angioplastia transluminal percutânea (ATP) pode nem sempre estar disponível em alguns hospitais no país e, em doentes mais jovens, como no caso do paciente aqui relatado, a técnica aberta tem melhor resultado a longo prazo. Além disso, a recanalização da artéria ilíaca externa, algumas vezes associada a doença da artéria femoral comum, pode ser extremamente complexa^1^^,^ 2. Quando a ATP das lesões ilíacas não é aceitável, a cirurgia convencional é o procedimento mais adequado a ser realizado^10^^,^ 11.

No caso aqui apresentado, a opção pelo tratamento cirúrgico teve como principal motivo a extensão da doença oclusiva, que acometia toda a transição ilíaco-femoral. A escolha do acesso cirúrgico para a anastomose proximal, utilizando a artéria ilíaca externa esquerda como artéria doadora, foi devido aos maiores índices de infecção de prega inguinal que temos em nosso serviço, talvez relacionados ao número de comorbidades e ao baixo nível socioeconômico de nossos pacientes^8^. A infecção de prega inguinal após procedimentos vasculares pode alcançar taxas entre 3 e 44%, segundo a literatura, com potenciais desfechos graves, como aumento de mortalidade e perda de membro^9^^,^ 12. Em relação ao substituto escolhido, nossa preferência foi pelo material venoso autógeno de membro superior, devido à maior compatibilidade de diâmetros anastomóticos e à baixa taxa de infecção^13^. No caso aqui apresentado, a escolha da composição venosa de veia de membro superior deu-se devido à inadequação da veia safena magna, ao espessamento venoso em topografia cubital, e à larga experiência de nosso serviço no uso do substituto venoso de membros superiores. A devalvulação do substituto venoso é a preferência do nosso serviço, pois preserva o padrão anatômico das artérias nativas em relação aos seus diâmetros proximal e distal, permitindo anastomoses mais compatíveis, além de manter a fasicidade arterial no enxerto.

A artéria descendente lateral femoral, conhecida pelo seu papel importante na colaterarização supra e infragenicular em oclusões femoropoplíteas, através da comunicação natural com as artérias geniculares, foi a única opção viável encontrada como artéria receptora para a derivação arterial. Seu uso ainda não foi descrito na literatura, mas procedimentos similares com artérias vicariantes alternativas, como a artéria genicular descendente e as artérias geniculares surais, constituem uma boa opção de derivação nos casos em que os substitutos são restritos ou mesmo naqueles em que não encontramos artérias tronculares receptoras adequadas^7^^,^ 14.

Como crítica à nossa conduta frente a esse procedimento, poderíamos citar a possibilidade de tratamento híbrido com ATP da lesão de ilíaca comum direita associada à derivação arterial para artéria anastomótica magna, evitando assim o enxerto cruzado. Porém, não dispúnhamos de material endovascular adequado no momento da internação.

Derivações e substitutos alternativos, utilizados normalmente em casos de exceção, podem e devem fazer parte do arsenal terapêutico vascular, podendo contribuir nos casos em que a cirurgia endovascular ainda não nos permite alcançar bons resultados^4^^,^ 5 ^,^ 7 ^,^ 15 ^,^ 16.
